# High expression of fibroblast‐activating protein is a prognostic marker in non‐small cell lung carcinoma

**DOI:** 10.1111/1759-7714.14579

**Published:** 2022-07-11

**Authors:** Naoki Yanagawa, Mayu Sugai, Shunsuke Shikanai, Ryo Sugimoto, Mitsumasa Osakabe, Noriyuki Uesugi, Hajime Saito, Makoto Maemondo, Tamotsu Sugai

**Affiliations:** ^1^ Department of Molecular Diagnostic Pathology Iwate Medical University Shiwa‐gun Japan; ^2^ Department of Thoracic Surgery Iwate Medical University Shiwa‐gun Japan; ^3^ Division of Pulmonary Medicine, Department of Internal Medicine Iwate Medical University Shiwa‐gun Japan

**Keywords:** fibroblast‐activating protein, immunohistochemistry, non‐small cell lung carcinoma, prognosis

## Abstract

**Background:**

Fibroblast‐activating protein (FAP) is expressed in cancer‐associated fibroblasts (CAFs) in many human carcinomas and in some types of carcinoma cells. Here, we examined the proportion of FAP protein expression in non‐small cell lung carcinoma (NSCLC) and investigated the correlation of FAP expression with clinicopathological background.

**Methods:**

In total, 344 NSCLC tissues were examined. Tissue microarrays were constructed, and FAP expression was analyzed using immunohistochemistry. The status of FAP expression in tumor cells and CAFs was correlated with clinicopathological background, molecular features, and patient outcomes.

**Results:**

A total of 280 patients (81.4%) had low FAP expression, and 64 patients (18.6%) had high FAP expression in tumor cells. In CAFs, 230 patients (66.9%) had low FAP expression, and 114 patients (33.1%) had high FAP expression. In multivariate analyses, high FAP expression in tumor cells was an independent predictive factor of both overall survival (OS; hazard ratio [HR] = 2.57, 95% confidence interval [CI]: 1.49–4.42, *p* < 0.001) and recurrence‐free survival (RFS; HR = 2.13, 95% CI: 1.38–3.29, *p* < 0.001). Based on combinations of FAP expression in tumor cells and CAFs, patients with Low^T^/Low^CAFs^ had better OS and RFS than did those in the other subgroups. By contrast, patients with High^T^/High^CAFs^ had poor OS and RFS compared with those in the other subgroups.

**Conclusions:**

Overall, FAP expression in tumor cells and the combination FAP expression in tumor cells and CAFs were strongly associated with patient survival and may be useful predictive biomarkers for patient outcomes in NSCLC.

## Graphical abstract

A total of 280 patients (81.4%) had low FAP expression, and 64 patients (18.6%) had high FAP expression in tumor cells. In CAFs, 230 patients (66.9%) had low FAP expression, and 114 patients (33.1%) had high FAP expression. In multivariate analyses, high FAP expression in tumor cells was an independent predictive factor of both overall survival (OS; HR = 2.57, 95% CI: 1.49–4.42, *p* < 0.001) and recurrence‐free survival (RFS; HR = 2.13, 95% CI: 1.38–3.29, *p* < 0.001).

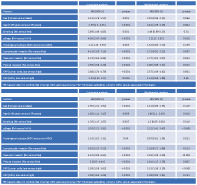

## INTRODUCTION

Lung cancer (LC) is the leading cause of cancer‐related death in developed countries.^1^, ^2^, ^3^ Non‐small cell lung carcinoma (NSCLC) accounts for approximately 85% of all LC cases. Although multidisciplinary therapy has improved outcomes in patients with LC, most patients are diagnosed at an advanced stage, and the 5‐year survival rate is only approximately 18%.^1^, ^2^, ^3^ Therefore, there is an urgent need to explore novel prognostic markers and therapeutic targets for LC.

Fibroblast‐activating protein (FAP; also known as seprase), a cell surface glycoprotein belonging to the serine protease family, is a 170‐kDa dimer that is catalytically active and has dipeptidase and gelatinase activities.^4^ FAP is expressed in reactive fibroblasts in the context of chronic inflammation and liver cirrhosis,^5^ in healing wounds,^6^ during embryonic development,^7^ and in cancer‐associated fibroblasts (CAFs) in many types of cancers in humans.^8^, ^9^, ^10^, ^11^ Cancer cells interact with surrounding stromal cells via complex mechanisms, making up the tumor microenvironment (TME).^12^ Within the last decade, the TME has been shown to be important for the proliferation, invasion, metastasis, and chemoresistance of cancer cells.^13^, ^14^, ^15^ One fundamental type of stromal cell is CAFs. As a marker of CAFs, FAP enhances stromal cell proliferation and invasiveness, mediates apoptosis,^10^, ^12^, ^13^ and is closely correlated with poor prognosis in various types of tumors, including NSCLC.^12^ Interestingly, FAP is also expressed in carcinoma cells of the stomach,^16^ colorectum,^17^ breast,^18^ uterine cervix,^19^ and pancreas^4^ and has been reported to be correlated with prognosis. Although some reports have described the relationships between FAP expression in CAFs from patients with NSCLC and prognosis, the association of FAP expression in NSCLC tissues with prognosis has not yet been elucidated.

Accordingly, in this study, we examined FAP protein expression in NSCLC and investigated correlations with clinicopathological factors, including patient outcomes.

## METHODS

### Patients

A retrospective review of a prospectively maintained surgical database was performed to identify patients who underwent primary LC resection with curative intent from 2015 to 2017. The histopathological diagnosis was made according to the eighth edition of the TNM Classification of the Union for International Cancer Control and the 2015 World Health Organization classification.^20^, ^21^ Patients were excluded from the current evaluation if they underwent chemotherapy or radiotherapy before surgery, underwent incomplete resection, had multiple primary lung cancers, or had incomplete follow‐up data. Finally, 344 patients with NSCLC were examined. The mean follow‐up period was 44.3 months (range, 4.2–80.8 months). This study was approved by the Institutional Review Board of Iwate Medical University (approval no. MH2020‐163) and was conducted according to the principles of the Declaration of Helsinki. Written informed consent was waived because this was a retrospective study, the patient data remained anonymous, and an opt‐out approach was used.

### Preparation of tissue samples and tissue microarrays (TMAs)

In total, 344 formalin‐fixed, paraffin‐embedded samples from consecutive resected NSCLC collected from 2015 to 2017 were used for the preparation of TMAs. We searched for the representative tumor area (the area of predominant subtypes as for adenocarcinoma) and the area of proliferation of spindle‐shaped cells in stroma. Then, we set up the area which fills with two criterions as previously stated and arrayed a cylindrical 3‐mm tissue core from the corresponding paraffin blocks into a recipient block using a tissue arrayer (KIN‐2; Azumaya). Hematoxylin and eosin staining was used to evaluate both tumor cells and CAFs in each TMA specimen.

### Evaluation of FAP protein expression

**FIGURE 1:**
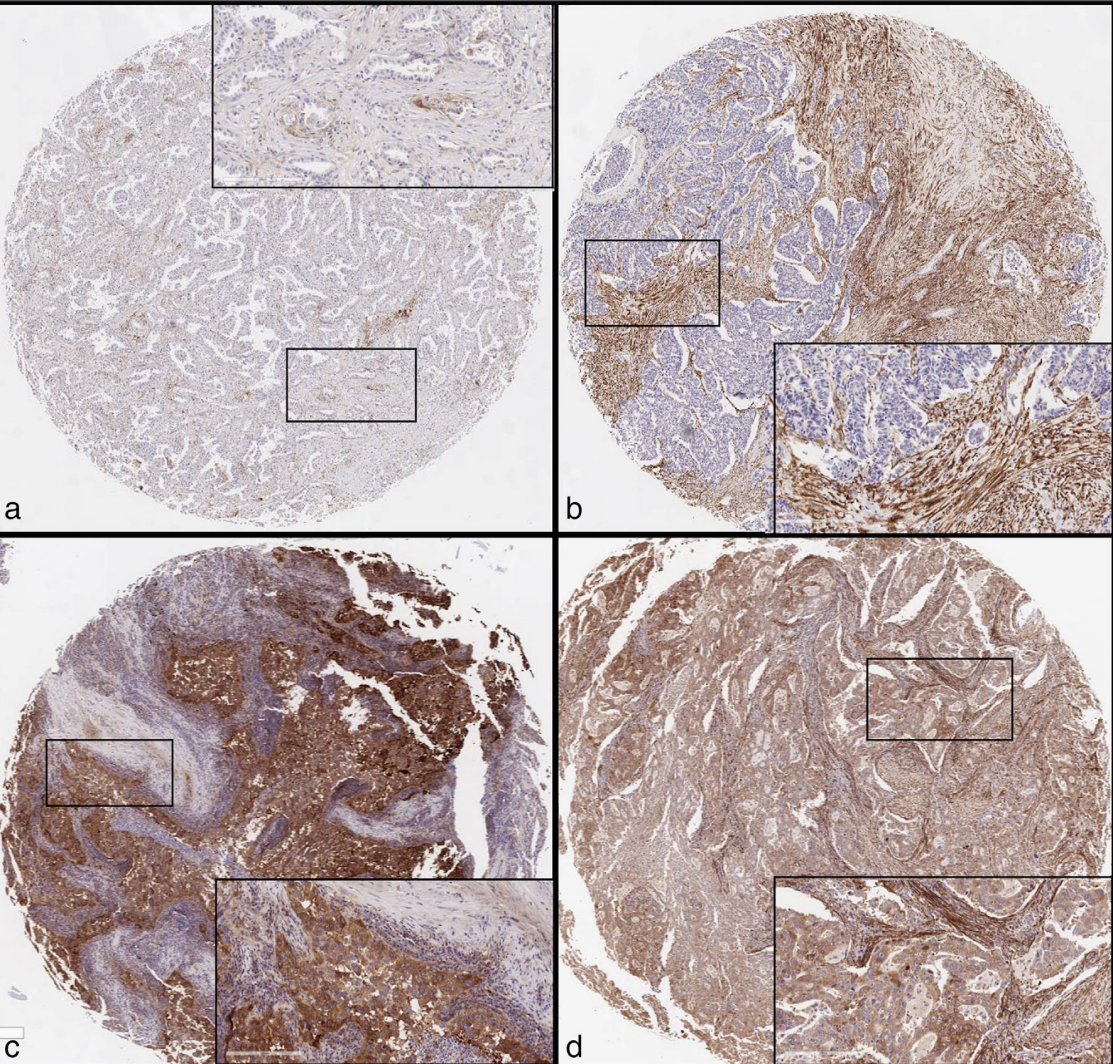
Representative images of immunohistochemical expression of fibroblast‐activating protein (FAP) in tumor cells and cancer‐associated fibroblasts (CAFs). (a) Low expression in both tumor cells and CAFs. (b) Low expression in tumor cells and high expression in CAFs. (c) High expression in tumor cells and low expression in CAFs. (d) High expression in both tumor cells and CAFs. Insets show high power view images, respectively

FAP protein expression was examined by immunohistochemistry. TMA blocks were sliced into 4‐μm‐thick sections, deparaffinized, and stained for FAP (Abcam; cat. no. EPR20021) using a DAKO Autostainer Universal Staining System (Dako). Two pathologists (N.Y. and N.U.) evaluated the slides. FAP protein expression was scored for both extent of immunopositivity and intensity, as previously described, with modifications.^4^, ^10^ Tumor cells and CAFs were evaluated separately. The extent of immunopositivity was semiquantified as follows: 0% (score 0), 1–10% (score 1), 11%–50% (score 2), and 51%–100% (score 3). The intensity was classified into 4 categories as follows: negative (score 0), weak (score 1), moderate (score 2), and strong (score 3). The sum of the two scores was used as the final score. A final score less than 3 was defined as low expression (including total score 0), and that of 3 or more was defined as high expression. Representative staining images are shown in Figure 1.

### Immunohistochemical analysis of anaplastic lymphoma kinase (ALK), p53, and programmed cell death‐1 ligand (PD‐L1)

We performed immunohistochemical staining using antibodies against ALK (clone D5F3; Roche) and p53 (clone DO‐7; Roche) in the TMAs. The immunohistochemical results were evaluated as follows: strong granular cytoplasmic staining of ALK in tumor cells (any percentage) was defined as ALK positivity; staining of p53 in greater than or equal to 10% of tumor cells was defined as p53 positivity. For PD‐L1, TMAs were stained for PD‐L1 using PD‐L1 IHC 22C3 pharmDx assays (Agilent Technologies, Inc.) and an Autostainer Link 48 using an automated staining protocol. If the membrane of the tumor cells was stained, the cells were considered positive for PD‐L1 protein expression. The tumor proportion score (TPS) of PD‐L1 in tumor cells was defined as the percentage of PD‐L1‐positive tumor cells in the TMA tumor sections. If the TPS was greater than or equal to 1%, the section was defined as having positive expression.

### Epidermal growth factor receptor (EGFR) gene mutation analysis

*EGFR* mutations were detected using real‐time polymerase chain reaction (SRL, Tokyo, Japan).

### Statistical analysis

Statistical comparisons were performed using χ^2^ tests or Fisher's exact tests, as appropriate. Recurrence‐free survival (RFS) and overall survival (OS) were analyzed using the Kaplan–Meier method, and differences in variables were calculated using log‐rank tests. RFS was defined as the time from surgery to recurrence, death, or the last follow‐up. OS was defined as the time from surgery to death or the last follow‐up. The last follow‐up observation was censored if the patient was alive or lost to follow‐up. A multivariate survival analysis was performed using the Cox proportional hazards model. All statistical analyses were performed using EZR (Saitama Medical Center, Jichi Medical University, Saitama, Japan), a modified version of R commander (R foundation for Statistical Computing) designed to add functions frequently used in biostatistics.^22^ Results with *p‐*values less than 0.05 were considered statistically significant.

## RESULTS

### Expression status of FAP protein in tumor cells and CAFs

Scoring of FAP expression in tumor cells and CAFs is summarized in Table 1. Briefly, 280 patients (81.4%) had low FAP expression, and 64 patients (18.6%) had high FAP expression in tumor cells (Table 1). In CAFs, 230 patients (66.9%) had low FAP expression, and 114 patients (33.1%) had high FAP expression (Table 2). The correlation between FAP expression in tumor cells and FAP expression in CAFs is shown in Table 3. The numbers (percentages) of the combinations of low expression in tumor cells/low expression in CAFs (Low^T^/Low^CAFs^), high expression in tumor cell/low expression in CAFs (High^T^/Low^CAFs^), low expression in tumor cell/high expression in CAFs (Low^T^/High^CAFs^), and high expression in tumor cell/high expression in CAFs (High^T^/High^CAFs^) were as follows: 198 (57.6%), 32 (9.3%), 82 (23.8%), and 32 (9.3%), respectively.

**TABLE 1:** Scoring of fibroblast‐activating protein (FAP) expression in tumor cells

	% Positive			
Tumor cells	0 (0%)	1 (1–10%)	2 (11–50%)	3 (> 50%)
0 (Negative)	Score 0 (246)	‐	‐	‐
1 (Weak)	‐	Score 2 (34)	Score 3 (4)	Score 4 (1)
2 (Moderate)	‐	Score 3 (10)	Score 4 (24)	Score 5 (6)
3 (Strong)	‐	Score 4 (2)	Score 5 (8)	Score 6 (9)

**TABLE 2:** Scoring of fibroblast‐activating protein (FAP) expression in CAFs

	% Positive			
CAFs	Score 0 (0%)	1 (1–10%)	2 (11%–50%)	3 (> 50%)
0 (Negative)	Score 0 (117)	‐	‐	‐
1 (Weak)	‐	Score 2 (113)	Score 3 (15)	Score 0
2 (Moderate)	‐	Score 3 (16)	Score 4 (35)	Score 5 (10)
3 (Strong)	‐	Score 4 (0)	Score 5 (16)	Score 6 (22)

Abbreviation: CAFs, cancer‐associated fibroblasts.

**TABLE 3:** Correlation between fibroblast‐activating protein (FAP) expression in tumor cells and CAFs

	Low^CAFs^ (230)	High^CAFs^ (114)
Low^T^ (280)	198 (57.6%)	82 (23.8%)
High^T^ (64)	32 (9.3%)	32 (9.3%)

Abbreviations: CAFs, cancer‐associated fibroblasts; T, tumor cells.

### Patient, clinicopathological, and molecular characteristics

Patient, clinicopathological, and molecular characteristics are summarized in Table 4. Tumors from 192 men (55.8%) and 152 women (44.2%), with a median age of 69.2 years (range, 40–87 years), were examined. Of the 344 patients, 162 (47.1%) and 182 (57.9%) were less than or equal to 69 years old and greater than or equal to 70 years old, respectively, and 135 (39.2%) were nonsmokers. Regarding pathological stage, 231 patients (67.2%) were classified as stage 0/I, 45 patients (13.1%) were classified as stage II, and 68 patients (19.8%) were classified as stage III. Histopathologically, 260 tumors (75.6%) were classified as adenocarcinoma, 64 tumors (18.6%) were classified as squamous cell carcinoma (SCC), and 20 tumors (5.8%) were classified as other histological subtypes. Detailed predominant subtypes of adenocarcinoma and other histological subtypes are shown in supplementary Table S1. Briefly, high FAP expression of both tumor cells and CAFs was frequently found in the patients with solid predominant adenocarcinoma (53.8% in tumor cells and 61.5% in CAFs, respectively) than other subtypes of adenocarcinoma. Lymphocytic invasion was found in 44 patients (12.8%), vascular invasion was found in 94 patients (27.3%), and pleural invasion was found in in 84 patients (24.4%). *EGFR* mutation analysis was performed in 243 patients, and 114 (46.9%) of these patients had *EGFR* mutations. p53 and PD‐L1 immunohistochemical expression was positive in 73 (32.8%), and 39 (16.6%) patients, respectively. Detailed *EGFR* mutation, ALK immunohistochemistry and adjuvant therapy are shown in supplementary Table S1.

**TABLE 4:** Relationships of FAP expression in tumor cells and CAFs with clinicopathological and molecular characteristics

	FAP expression (tumor cells)			FAP expression (CAFs)		
	Low	High		Low	High	
Variables	(n = 280, 81.4%)	(n = 64, 18.6%)	*p*‐value	(n = 230, 66.9%)	(n = 114, 33.1%)	*p*‐value
Sex						
Male (192)	156 (81.2)	36 (18.8)	0.992	112 (58.3)	80 (41.7)	< 0.001
Female (152)	124 (81.6)	28 (18.4)		118 (77.6)	34 (22.4)	
Age						
≤ 69 years (162)	139 (85.8)	23 (14.2)	0.065	114 (70.4)	48 (29.6)	0.234
≥ 70 years (182)	141 (77.5)	41 (22.5)		116 (63.7)	66 (36.3)	
Smoking						
No (135)	110 (81.5)	25 (18.5)	0.996	109 (80.7)	26 (19.3)	< 0.001
Yes (209)	170 (81.3)	39 (18.7)		121 (57.9)	88 (42.1)	
Stage						
0‐I (231)	195 (84.4)	36 (15.6)	0.056 (0–I vs. II–III)	173 (74.9)	58 (25.1)	< 0.001 (0–I versus II–III)
II (45)	35 (77.8)	10 (22.2)		23 (51.1)	22 (48.9)	
III (68)	50 (73.5)	18 (26.5)		34 (50)	34 (50)	
Histology						
ADC (260)	208 (80)	52 (20)	0.372 (ADC vs. SCC)	194 (74.6)	66 (25.4)	< 0.001 (ADC versus SCC)
SCC (64)	55 (85.9)	9 (14.1)		24 (37.5)	40 (62.5)	
Others (20)	17 (85)	3 (15)		12 (60)	8 (40)	
Lymphocytic invasion						
No (300)	248 (82.7)	52 (17.3)	0.169	210 (70)	90 (30)	0.002
Yes (44)	32 (72.7)	12 (27.3)		20 (45.5)	24 (54.5)	
Vascular invasion						
No (250)	212 (84.8)	38 (15.2)	0.013	190 (76)	60 (24)	< 0.001
Yes (94)	68 (72.3)	26 (27.7)		40 (42.6)	54 (57.4)	
Pleural invasion						
No (260)	216 (83.1)	44 (16.9)	0.212	185 (71.2)	75 (28.8)	0.004
Yes (84)	64 (76.2)	20 (23.8)		45 (53.6)	39 (46.4)	
*p53* IHC						
Negative (231)	193 (83.5)	38 (16.5)	0.187	172 (74.5)	59 (25.5)	< 0.001
Positive (113)	87 (77)	26 (23)		58 (51.3)	55 (48.7)	
PD‐L1 IHC						
Negative (287)	248 (86.4)	39 (13.6)	< 0.001	209 (72.8)	78 (27.2)	< 0.001
Positive (57)	32 (56.1)	25 (43.9)		21 (3.8)	36 (63.2)	

Abbreviations: ADC, adenocarcinoma; CAFs, cancer‐associated fibroblasts; FAP, fibroblast‐activating protein; IHC, immunohistochemistry; PD‐L1, programmed death ligand 1; SCC, squamous cell carcinoma.

### Relationship of FAP expression in tumor cells and CAFs with clinicopathological and molecular characteristics

The associations of clinicopathological and molecular characteristics with FAP expression in tumor cells are shown in Table 4. High FAP expression in tumor cells was more frequently found in patients with vascular invasion (*p* = 0.013) and positive PD‐L1 expression (*p* < 0.001).

The associations of clinicopathological and molecular characteristics with FAP expression in CAFs are also shown in Table 4. High FAP expression in CAFs was more frequently found in men (*p* < 0.001), smokers (*p* < 0.001), and patients with more advanced stage disease (*p* < 0.001), SCC (*p* < 0.001), lymphocytic invasion (*p* = 0.002), vascular invasion (*p* < 0.001), pleural invasion (*p* = 0.004), positive p53 expression (*p* < 0.001), and positive PD‐L1 expression (*p* < 0.001).

### Univariate and multivariate analyses based on OS and RFS

The mean follow‐up period was 44.3 months (range, 4.2–80.8 months); 66 of the 344 patients died during the follow‐up period. Of these, 43 died as a result of cancer recurrence, and the remaining 23 died of other causes. Of the 278 patients who were alive at the time of analysis, 44 had recurrent disease, and 234 had no evidence of disease.

The 3‐year OS rate in all patients was 84.9%. Univariate analysis revealed that sex, patient age, smoking, pathological stage, histological subtype, lymphocytic invasion, vascular invasion, pleural invasion, FAP expression in tumor cells, and FAP expression in CAFs were significant prognostic factors (Table 5). Multivariate analysis showed that patient age, pathological stage, vascular invasion, pleural invasion, and FAP expression in tumor cells were independent prognostic factors (OS; hazard ratio [HR] = 2.57, 95% confidence interval [CI]: 1.49–4.42, *p* < 0.001) (Table 5).

**TABLE 5:** Univariate and multivariate analyses based on overall survival

	Univariate analysis		Multivariate analysis	
Factors	HR (95% CI)	*p*‐value	HR (95% CI)	*p*‐value
Sex (female vs. male)	3.13 (1.76–5.57)	0.001	2.01 (0.96–4.21)	0.066
Age (≤ 69 years vs. ≥70 years)	2.59 (1.5–4.45)	< 0.001	2.44 (1.39–4.29)	0.002
Smoking (no vs. yes)	2.69 (1.49–4.85)	0.001	1.49 (0.69–3.23)	0.31
pStage (0–I vs. II–III)	4.05 (2.47–6.63)	< 0.001	2 (1.12–3.55)	0.018
Histological subtype (ADC vs. non‐ADC)	2.1 (1.24–3.39)	0.005	1.52 (0.85–2.72)	0.159
Lymphocytic invasion (no vs. yes)	4.31 (2.59–7.15)	< 0.001	1.71 (0.91–3.22)	0.097
Vascular invasion (no vs. yes)	4.27 (2.63–6.94)	< 0.001	1.77 (1.02–3.07)	0.043
Pleural invasion (no vs. yes)	2.59 (1.58–4.26)	< 0.001	1.86 (1.09–3.17)	0.023
FAP (tumor cells, low vs. high)	2.88 (1.74–4.79)	< 0.001	2.57 (1.49–4.42)	0.001
FAP (CAFs, low vs. high)	2.34 (1.45–3.8)	<0.001	1.13 (0.68–1.88)	0.65

Abbreviations: ADC, adenocarcinoma; CAFs, cancer‐associated fibroblasts; CI, confidential interval; FAP, fibroblast‐activating protein; HR, hazard ratio.

The 3‐year RFS rate in all patients was 71.7%. Univariate analysis revealed that sex, patient age, smoking, pathological stage, histological subtype, lymphocytic invasion, vascular invasion, pleural invasion, FAP expression in tumor cells, and FAP expression in CAFs were significant prognostic factors for recurrence (Table 6). Multivariate analysis showed that patient age, pathological stage, vascular invasion, pleural invasion, and FAP expression in tumor cells were independent predictive factors for recurrence (RFS; HR = 2.13, 95% CI: 1.38–3.29, *p* < 0.001)(Table 6).

**TABLE 6:** Univariate and multivariate analyses based on recurrence‐free survival

	Univariate analysis		Multivariate analysis	
Factors	HR (95% CI)	*p*‐value	HR (95% CI)	*p*‐value
Sex (female vs. male)	1.95 (1.31–2.92)	< 0.001	1.51 (0.89–2.55)	0.119
Age (≤ 69 years vs. ≥70 years)	1.68 (1.11–2.47)	0.009	1.66 (1.1–2.49)	0.016
Smoking (no vs. yes)	1.76 (1.17–2.67)	0.007	1.2 (0.69–2.08)	0.516
pStage (0–I vs. II–III)	3.97 (2.71–5.82)	< 0.001	2.22 (1.41–3.47)	< 0.001
Histological subtype (ADC vs. non‐ADC)	1.53 (1.02–2.31)	0.04	0.97 (0.61–1.54)	0.921
Lymphocytic invasion (no vs. yes)	3.62 (2.37–5.52)	< 0.001	1.18 (0.71–1.98)	0.523
Vascular invasion (no vs. yes)	4.52 (3.09–6.61)	< 0.001	2.18 (1.39–3.39)	<0.001
Pleural invasion (no vs. yes)	3 (2.03–4.44)	< 0.001	1.81 (1.17–2.78)	0.007
FAP (tumor cells, low vs. high)	2.39 (1.58–3.62)	< 0.001	2.13 (1.38–3.29)	< 0.001
FAP (CAFs, low vs. high)	2.38 (1.63–3.46)	< 0.001	1.28 (0.85–1.91)	0.231

Abbreviations: ADC, adenocarcinoma; CAFs, cancer‐associated fibroblasts; CI, confidential interval; FAP, fibroblast‐activating protein; HR, hazard ratio.

### OS and RFS according to combinations of FAP expression in tumor cells and CAFs

**FIGURE 2:**
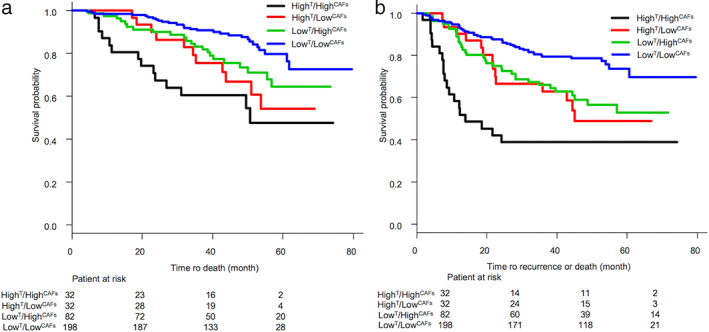
Overall survival (OS) curve (a) and recurrence‐free survival (RFS) curve (b) according to combinations of fibroblast‐activating protein (FAP) expression in tumor cells and cancer‐associated fibroblasts (CAFs). Patients with Low^T^/Low^CAFs^ had better OS and RFS than did those in the other subgroups. By contrast, patients with High^T^/High^CAFs^ had poor OS and RFS rates compared with those in the other subgroups. Superscript T means tumor cells. In addition, patients with High^T^/Low^CAFs^ and patients with Low^T^/High^CAFs^ had almost the same OS and RFS, and their survival curves were located at between survival curve of patients with Low^T^/Low^CAFs^ and survival curve of patients with High^T^/High^CAFs^

OS and RFS curves according to combinations of FAP expression in tumor cells and CAFs are shown in Figure 2a (OS) and Figure 2b (RFS). Patients with Low^T^/Low^CAFs^ had a better OS and RFS than those in the other subgroups. In contrast, patients with High^T^/High^CAFs^ had a poor OS and RFS compared with those in the other subgroups. In addition, patients with High^T^/Low^CAFs^ and patients with Low^T^/High^CAFs^ had almost the same OS and RFS, and their survival curves were located at  between survival curve of patients with Low^T^/Low^CAFs^ and survival curve of patients with High^T^/High^CAFs^.

## DISCUSSION

Previous cancer research has mainly focused on cancer cells. However, in the last 20 years, the stromal cells surrounding cancer cells have also been shown to be important factors for cancer development. Cancer cells interact in complex ways with surrounding stromal cells, making up the TME.^12^, ^23^ The stromal cells surrounding cancer cells include CAFs, endothelial cells, and inflammatory cells.^12^, ^24^ CAFs are thought to be derived from various other types of cells, including resident fibroblasts, bone marrow‐derived progenitor cells, or even epithelial carcinoma cells undergoing the epithelial‐to‐mesenchymal transition.^25^, ^26^ CAFs are activated fibroblasts in a cancer microenvironment and have been shown to interact with carcinoma cells and other components of the microenvironment, including immune cells and blood vessels, thereby influencing the biological behaviors of carcinoma cells, such as their proliferation, migration, and invasion.^12^, ^25^, ^26^ As a marker of CAFs, FAP enhances stromal cell proliferation and invasiveness, affects cell apoptosis, and is closely correlated with poor prognosis in various types of tumors, including NSCLC.

Interestingly, FAP expression was found not only in CAFs but also in LC cells for the first time. In other cancers, FAP expression has been found in cancer cells of the stomach,^16^ colorectum,^17^ breast,^18^ ovaries,^24^ uterine cervix,^19^ and pancreas^4^ and has been shown to be correlated with histological grade, invasion, and metastatic progression in some cancers. In our study, more advanced stage and vascular invasion were associated with high expression of FAP in cancer cells. Shi et al. suggested that pancreatic ductal adenocarcinoma cells may contribute directly to stroma desmoplasia through an autocrine mechanism involving FAP protein.^4^ This same mechanism may function in LC cells and surrounding stromal cells. Furthermore, Shi et al. also reported that higher FAP expression in pancreatic cancer cells is associated with worse clinical outcomes, consistent with our current findings.^4^ In particular, FAP expression in tumor cells was an independent prognostic factor and independent predictive factor for recurrence. Therefore, FAP expression in cancer cells may be a useful predictive biomarker for patient outcomes.

FAP expression in CAFs has been described in some types of cancers. To date, three studies have reported FAP expression in CAFs from patients with NSCLC.^11^, ^27^, ^28^ All these studies describe the relationship of FAP expression with clinicopathological factors. Liao et al. reported that high expression of FAP may be correlated with poor tumor differentiation and that both increased FAP staining percentage and intensity were associated with worse OS in patients.^11^ Furthermore, Chen et al. showed that CAF density is significantly associated with lymph node metastasis and that there is a negative correlation between CAF density and survival.^27^ In contrast, Kilvaer et al. reported that the presence of FAP‐1‐expressing CAFs is an indicator of positive outcomes in patients with NSCLC‐SCC (squamous cell carcinoma).^28^ In our univariate analysis, FAP expression in CAFs was found to be a significant prognostic factor and predictive factor of recurrence; however, this factor was not significant in multivariate analysis. Therefore, evaluation of FAP expression in CAFs alone may not be sufficient. In our study, High^T^/High^CAFs^ was associated with significantly poorer OS rates than Low^T^/High^CAFs^ and Low^T^/Low^CAFs^, and both High^T^/Low^CAFs^ and Low^T^/High^CAFs^ were associated with significantly poorer OS rates than Low^T^/Low^CAFs^. Thus, the combination of FAP expression in tumor cells and CAFs should be assessed.

Recently, immunotherapy using immune checkpoint antibodies targeting PD‐1 (programmed cell death‐1/PD‐L1 [programmed cell death‐1 ligand]) has been shown to improve outcomes in patients with various malignant tumors.^29^, ^30^ Pembrolizumab is a humanized monoclonal antibody targeting PD‐1 that exerts antitumor activity in advanced NSCLC; its therapeutic effect is closely related to PD‐L1 expression in cancer cells, and PD‐L1 protein expression has been suggested to be a predictive biomarker of the response to immunotherapy.^31^ In our study, both FAP expression in tumor cells and FAP expression in CAFs were strongly correlated with PD‐L1 expression. Although further analyses are required, these findings suggested that FAP expression may be a predictive biomarker of the efficacy of immune checkpoint inhibitors. Further studies are needed to assess the involvement of the TME, including immune‐related inflammatory cells.

This study had some limitations. First, we used TMAs rather than large tissue sections. Because TMAs may not always be representative of the entire tumor, heterogeneity of FAP expression is a major issue. Second, this was a retrospective study performed at a single institution; thus, the possibility of bias cannot be excluded.

In conclusion, our results showed that FAP expression in tumor cells and the combination of FAP expression in tumor cells and CAFs was strongly associated with patient survival. Thus, we suggest that FAP expression in these cells may be a useful predictive biomarker for clinical outcomes in patients with NSCLC.

## CONFLICT OF INTEREST

The authors declare that they have no conflicts of interest.

## Supporting information


**Table S1** Relationships of FAP expression in tumor cells and CAFs with clinicopathological and molecular characteristics.
