# Supplementary material for: High expression of fibroblast‐activating protein is a prognostic marker in non‐small cell lung carcinoma
Source: Thorac Cancer. 2022 Jul 11;13(16):2377–84. doi: 10.1111/1759-7714.14579 (PMC9376177; doi:10.1111/1759-7714.14579)
Supplement: Supplementary file 1 — Table S1 Relationships of FAP expression in tumor cells and CAFs with clinicopathological and molecular characteristics. [file TCA-13-2377-s001.docx]

Supplementary Table. Relationships of FAP expression in tumor cells and CAFs with clinicopathological and molecular characteristics

|  |  | FAP expression (tumor cells) |  |  | FAP expression (CAFs) |  |
| --- | --- | --- | --- | --- | --- | --- |
| Variables | Low | High | *p* value | Low | High | *p* value |
|  | (n = 280, 81.4%) | (n = 64, 18.6%) |  | (n = 230, 66.9%) | (n = 114, 33.1%) |  |
| ADC (260) | 208 (80) | 52 (20) | 0.372 (ADC versus SCC) | 194 (74.6) | 66 (25.4) | < 0.001 (ADC versus SCC) |
| Predominant subtypes |  |  |  |  |  |  |
| AIS (32) | 31 (96.8) | 1 (3.2) |  | 32 (100) | 0 (0) |  |
| MIA (35) | 30 (85.7) | 5 (14.3) |  | 32 (91.4) | 3 (8.6) |  |
| LPA (15) | 14 (93.3) | 1 (6.7) |  | 13 (86.7) | 2 (13.3) |  |
| APA (54) | 44 (81.5) | 10 (18.5) |  | 38 (70.4) | 16 (29.6) |  |
| PPA (76) | 56 (73.7) | 20 (26.3) |  | 49 (64.5) | 27 (35.5) |  |
| IMA (21) | 20 (95.2) | 1 (4.8) |  | 19 (90.5) | 2 (9.5) |  |
| MicPA (1) | 1 (100) | 0 (0) |  | 1 (100) | 0 (0) |  |
| SPA (26) | 12 (46.2) | 14 (53.8) |  | 10 (38.5) | 16 (61.5) |  |
| SCC (64) | 55 (85.9) | 9 (14.1) |  | 24 (37.5) | 40 (62.5) |  |
| Others (20) | 17 (85) | 3 (15) |  | 12 (60) | 8 (40) |  |
| ADSQ (4) | 4 (100) | 0 (0) |  | 2 (50) | 2 (50) |  |
| Carcinoid (4) | 4 (100) | 0 (0) |  | 4 (100) | 0 (0) |  |
| PC (4) | 2 (50) | 2 (50) |  | 1 (25) | 3 (75) |  |
| LCC (3) | 3 (100) | 0 (0) |  | 2 (66.7) | 1 (33.3) |  |
| ACC (2) | 2 (100) | 0 (0) |  | 2 (100) | 0 (0) |  |
| LCNEC (2) | 2 (66.7) | 1 (33.3) |  | 0 (0) | 2 (100) |  |
| MC (1) | 1 (100) | 0 (0) |  | 1 (100) | 0 (0) |  |
| *EGFR* mutation |  |  |  |  |  |  |
| No (129) | 102 (79.1) | 27 (20.9) | 0.633 (No versus Yes) | 91 (70.5) | 38 (29.5) | 0.712 (No versus Yes) |
| Yes (114) | 93 (81.6) | 21 (18.4) |  | 85 (74.6) | 29 (25.4) |  |
| Unknown (101) | 85 (84.2) | 16 (14.8) |  | 54 (53.5) | 47 (46.5) |  |
| *ALK* IHC |  |  |  |  |  |  |
| Negative (338) | 277 (82) | 61 (18) | 0.143 | 225 (66.6) | 113 (33.4) | 0.669 |
| Positive (6) | 3 (50) | 3 (50) |  | 5 (88.3) | 1 (16.7) |  |
| Adjuvant therapy |  |  |  |  |  |  |
| No (190) | 157 (82.6) | 33 (17.4) | 0.606 | 143 (75.3) | 47 (24.7) | < 0.001 |
| Yes (154) | 123 (79.9) | 31 (20.1) |  | 87 (56.5) | 67 (43.5) |  |
| FAP, fibroblast-activating protein; CAFs, cancer-associated fibroblasts; ADC, adenocarcinoma; AIS, adenocarcinoma in situ; MIA, minimally invasive adenocarcinoma; LPA, lepidic predominant adenocarcinoma; APA, acinar predominant adenocarcinoma; PPA, papillary predominant adenocarcinoma; IMA, invasive mucinous adenocarcinoma; MicPA, micropapillary predominant adenocarcinoma; SPA, solid predominant adenocarcinoma; SCC, squamous cell carcinoma; ADSQ, adenosquamous carcinoma; PC, pleomorphic carcinoma; LCC, large cell carcinoma; ACC, adenoid cystic carcinoma; LCNEC, large cell neuroendocrine carcinoma; MC, mucoepidermoid carcinoma; *EGFR*, epidermal growth factor receptor; ALK, anaplastic lymphoma kinase; Immunohistochemistry | | | | | | |
|  | | | | | |  |
